# The use of diuretics in heart failure with congestion: we can't judge a book by its cover

**DOI:** 10.1002/ehf2.12515

**Published:** 2019-10-16

**Authors:** Alberto Palazzuoli, Gaetano Ruocco, Stefania Paolillo, Pasquale Perrone Filardi

**Affiliations:** ^1^ Department of Internal Medicine, Cardiovascular Diseases Unit, Le Scotte Hospital University of Siena Viale Bracci n. 16 53100 Siena Italy; ^2^ Department of Advanced Biomedical Sciences Federico II University of Naples Naples Italy

## Abstract

Although congestion assessment is one of the major challenges in the management of acute heart failure (AHF), no specific Gguidelines have been previously published on this topic. A recent position paper from ESC HF society, focused attention on targeting therapy in relation to clinical congestion. However, in our opinion, the scarce correlation existing between wedge pressure with and traditional clinical signs is the fundamental concern in congestion detection. The two main characteristics of congestion are redistribution of blood volume from the systemic to the pulmonary district and intravascular fluid retention, and both of these are often employed with different significance. Another weakness could come from the varied congestion appearance in relation to the clinical scenario: the different pattern could vary in relation to prevalent cardiac dysfunction ([i.e. heart failure with reduced ejection fraction [(HFrEF]) vs. heart failure with preserved ejection fraction [(HFpEF)]), the Stevenson picture, and the history of recurrent or de novo HF. Thus, it could be worth distinguishing between central and peripheral congestion, in relation to the involved sites and organ damage. Our advice for a more effective diuretic dose is to obtain a better assessment of congestion by an integration between the clinical examination and a diagnostic algorithm taking into account echo and laboratory parameters. Consequently, the use of intravenous diuretics needs to be tailored in relation to the severity of congestion, the oral administration amount, and the different HF profiles.

We are very impressed by this position paper of the HFA group focusing attention on the demanding balance occurring between congestion and diuretics administration.^1^ Indeed, no specific guidelines have been previously published on this topic, although congestion assessment and its solution by current depletion treatment are two of the major challenges in the management of acute heart failure (AHF).^2^, ^3^ As emphasized in the paper, a specific clinical and diagnostic algorithm grading congestion does not exist. Similarly, a dose escalation scheme for the amount of loop diuretics is lacking, and the administration is often based on urine output and symptoms relief. Unfortunately, these two approaches have not yet been validated and are poorly related to prognosis. They depend, respectively, on renal perfusion, the pulmonary congestion timing course, and fluid intake during the observational period.^4^ Thus, a practical model assessing volume overload and grading congestion is imperative to tailor the best diuretic dose. Along these lines, we will describe some concepts in detail.

First of all, congestion is the ‘tip of the iceberg’ and the final effect of cardiac dysfunction leading to increased filling pressure transmitted backwards to the pulmonary circulation and central venous system.^5^ The interchangeable concept of volume overload and congestion should be addressed by taking into account the intravascular or interstitial compartment and the main site of fluid retention.^6^ Both items could fluctuate in relation to splanchnic and abdominal vessels capacitance, hepatic congestion and relative protein production, right site dysfunction, and central venous pressure. Moreover, the different congestion pattern and appearance could vary in relation to prevalent cardiac dysfunction [i.e. heart failure with reduced ejection fraction (HFrEF) vs. heart failure with preserved ejection fraction (HFpEF)], the Stevenson picture, and the history of recurrent or de novo HF.^3^, ^7^ The scarce correlation existing between invasive haemodynamic measurement of right atrial pressure or wedge pressure and traditional clinical signs could be explained by the wide AHF presentation, and each HF phenotype portends to a specific congestion pattern.^8^ In this framework, the diagnostic accuracy of congestion based on clinical signs, dyspnoea score, and chest radiography is prone to inter‐observer variability and is quite inaccurate. Therefore, congestion occurrence could be different in the pulmonary and systemic districts depending on right ventricular dysfunction, central venous hypertension, and HF timing course [again de novo vs. worsening decompensated heart failure (HF)]. Taken together, all these considerations emphasize the concept that we ‘can't judge a book by its cover’. Accordingly, the introduction of new diagnostic echo features, which are easily detectable in acute settings such as pulmonary b‐lines, E/e^1^ ratio, and cava vein collapse, could provide relevant information about pulmonary congestion, LV filling pressure, and venous hypertension, respectively. Although current features demonstrated a good prognostic impact in chronic patients, less is known in acute settings.^9^, ^10^ However, the proposed diagnostic scheme does not account for two other relevant parameters such as pulmonary artery systolic pressure (PASP) estimation by tricuspid regurgitation and right ventricle contractility measured by tricuspid annular plane systolic excursion (TAPSE). Both measurements are a mirror of ventricle‐pulmonary coupling and pulmonary vascular resistance.^11^, ^12^

The other imperative issues are which of these parameters are related to outcome in an acute setting and whether randomized studies demonstrated that a reduction of VCI, PASP, and E/e^1^ ratio after diuretic treatment is really associated with adverse event reduction. Thus, there is an impelling need to analyse all these aspects in a focused manner.

Regarding the euvolaemia determination, clinicians can benefit from some laboratory parameters: serial natriuretic peptide use could allow for a tailored therapy and better assessment of fluid retention. Despite this theoretical application, the last GUIDE‐IT trial did not demonstrate any benefit from BNP measurement.^13^ These findings raised some questions about the NP target for guiding management, the best timing for NP guided HF care, and the factors potentially confounding the use of NP during acute phases. Another parameter for potential application could be plasma osmolarity evaluated in association with haemoconcentration, urine concentration, and sodium level. Taken together, these features may become a marker for effective circulating volume and a surrogate for residual congestion detection.^14^

The final point we would like to underline is about the practical use of intravenous diuretics, which need to be tailored in relation to the severity of congestion, the previous oral administration, and the different HF profiles: it is plausible that congestion could differ in HFrEF, HFmEF, and HFpEF, similar to renal dysfunction occurrence and severity, because ‘one size doesn't fit all’. This requires a single approach in accordance with diuretic responsiveness, the main site of body fluid retention, and systemic pressure value (*Figure* *1*). The recommended dose escalation therapy based on 2.5 times the home dose may not be applied in all patients, and it could potentially lead to electrolyte imbalance, sudden pressure reduction, and worsening renal function.^15^ Similarly, the definition of a good response based on urine output >3–4 L/day seems overestimated. Indeed, this value depends on intravenous and oral fluid intake amount during the infusion diuretic period. We believe that a threshold of 3 L could be considered a good response assuming that these patients do not exceed 1 L of fluid intake, at least during the acute treatment.

**Figure 1:**
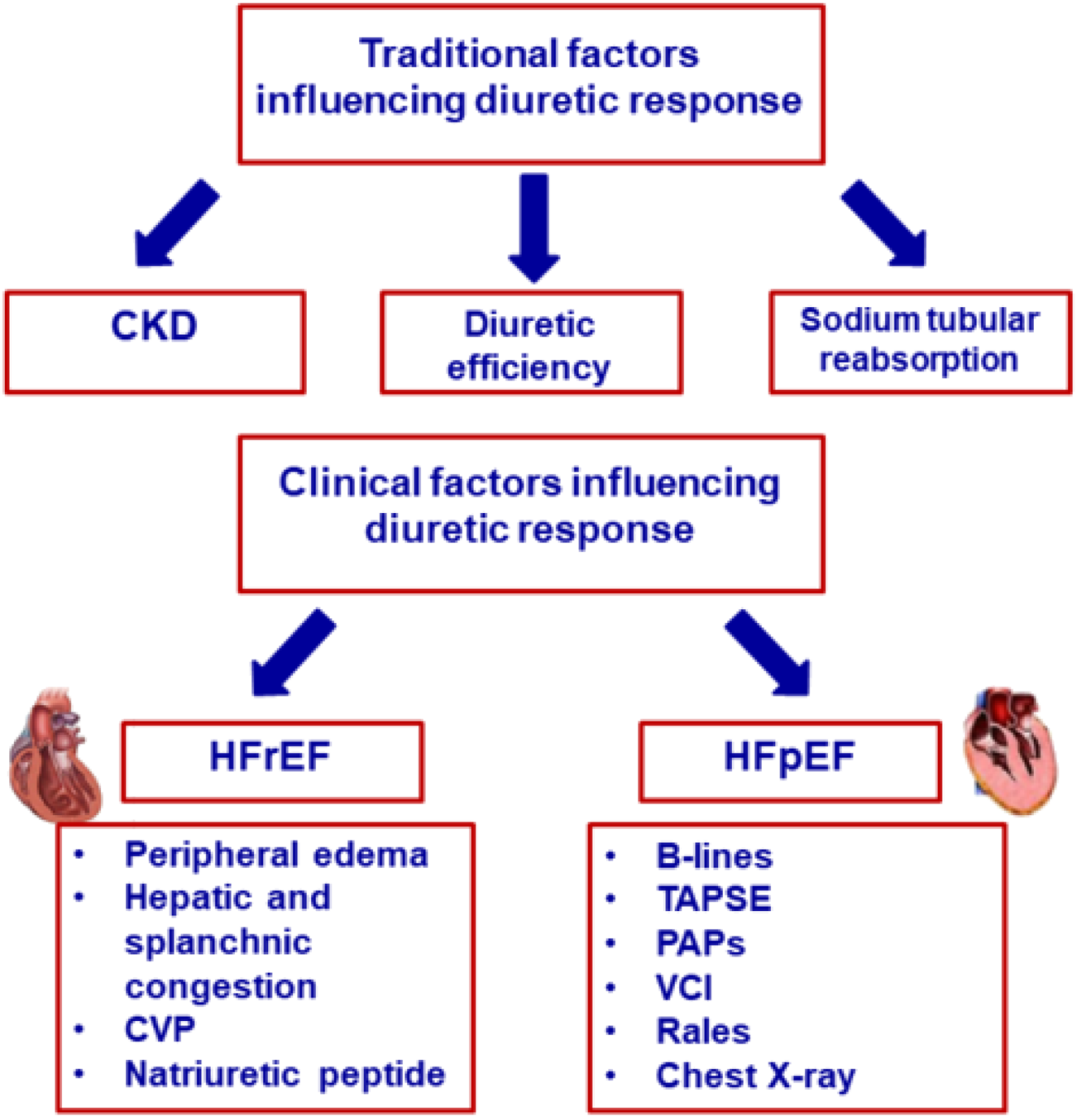
Determinants of diuretic response in HF patients.

In conclusion, we believe that the simple application of a clinical congestion score based only on symptoms and signs has limited value both for the euvolaemia assessment and for the diuretic therapy arrangement. Accordingly, some diagnostic tools should be added to the traditional evaluation, although most of these features have not yet demonstrated a prognostic impact. There is an impelling need to recognize different types of congestion in relation to the clinical picture and the prevalent pathophysiological mechanism sustaining acute HF. Clinical research evaluating both laboratory and echo features variation from baseline to discharge could improve the clinical course and treatment.

## Conflict of interest

None declared.

## References

[1] Mullens W , Damman K , Harjola VP , Mebazaa A , Brunner‐La Rocca HP , Martens P , Testani JM , Tang WHW , Orso F , Rossignol P , Metra M , Filippatos G , Seferovic PM , Ruschitzka F , Coats AJ . The use of diuretics in heart failure with congestion—a position statement from the Heart Failure Association of the European Society of Cardiology. Eur J Heart Fail 2019; 21: 137–155. 30600580 10.1002/ejhf.1369

[2] Ponikowski P , Voors AA , Anker SD , Bueno H , Cleland JG , Coats AJ , Falk V , González‐Juanatey JR , Harjola VP , Jankowska EA , Jessup M , Linde C , Nihoyannopoulos P , Parissis JT , Pieske B , Riley JP , Rosano GM , Ruilope LM , Ruschitzka F , Rutten FH , van der Meer P , Authors/Task Force Members , Document Reviewers . 2016 ESC Guidelines for the diagnosis and treatment of acute and chronic heart failure: The Task Force for the diagnosis and treatment of acute and chronic heart failure of the European Society of Cardiology (ESC). Developed with the special contribution of the Heart Failure Association (HFA) of the ESC. Eur J Heart Fail 2016; 18: 891–975. 27207191 10.1002/ejhf.592

[3] Gheorghiade M , Pang PS . Acute heart failure syndromes. J Am Coll Cardiol 2009; 53: 557–573. 19215829 10.1016/j.jacc.2008.10.041

[4] Palazzuoli A , Ruocco G , Ronco C , McCullough PA . Loop diuretics in acute heart failure: beyond the decongestive relief for the kidney. Crit Care 2015; 19: 296. 26335137 10.1186/s13054-015-1017-3 PMC4559070

[5] Gheorghiade M , Follath F , Ponikowski P , Barsuk JH , Blair JE , Cleland JG , Dickstein K , Drazner MH , Fonarow GC , Jaarsma T , Jondeau G , Sendon JL , Mebazaa A , Metra M , Nieminen M , Pang PS , Seferovic P , Stevenson LW , van Veldhuisen DJ , Zannad F , Anker SD , Rhodes A , McMurray JJ , Filippatos G , European Society of Cardiology , European Society of Intensive Care Medicine . Assessing and grading congestion in acute heart failure: a scientific statement from the acute heart failure committee of the heart failure association of the European Society of Cardiology and endorsed by the European Society of Intensive Care Medicine. Eur J Heart Fail 2010; 12: 423–433. 20354029 10.1093/eurjhf/hfq045

[6] Nijst P , Verbrugge FH , Grieten L , Dupont M , Steels P , Tang WHW , Mullens W . The pathophysiological role of interstitial sodium in heart failure. J Am Coll Cardiol 2015; 65: 378–388. 25634838 10.1016/j.jacc.2014.11.025

[7] Ambrosy AP , Bhatt AS , Gallup D , Anstrom KJ , Butler J , DeVore AD , Felker GM , Fudim M , Greene SJ , Hernandez AF , Kelly JP , Samsky MD , Mentz RJ . Trajectory of congestion metrics by ejection fraction in patients with acute heart failure (from the Heart Failure Network). Am J Cardiol 2017; 120: 98–105. 28479167 10.1016/j.amjcard.2017.03.249 PMC5471496

[8] Stevenson LW , Perloff JK . The limited reliability of physical signs for estimating hemodynamics in chronic heart failure. JAMA 1989; 261: 884–888. 2913385

[9] Pellicori P , Shah P , Cuthbert J , Urbinati A , Zhang J , Kallvikbacka‐Bennett A , Clark AL , Cleland JG . Prevalence, pattern and clinical relevance of ultrasound indices of congestion in outpatients with heart failure. Eur J Heart Fail 2019; 21: 904–916. 30666769 10.1002/ejhf.1383

[10] Palazzuoli A , Ruocco G , Beltrami M , Nuti R , Cleland JG . Combined use of lung ultrasound, B‐type natriuretic peptide, and echocardiography for outcome prediction in patients with acute HFrEF and HFpEF. Clin Res Cardiol 2018; 107: 586–596. 29532155 10.1007/s00392-018-1221-7

[11] Guazzi M , Bandera F , Pelissero G , Castelvecchio S , Menicanti L , Ghio S , Temporelli PL , Arena R . Tricuspid annular plane systolic excursion and pulmonary arterial systolic pressure relationship in heart failure: an index of right ventricular contractile function and prognosis. Am J Physiol Heart Circ Physiol 2013; 305: H1373–H1381. 23997100 10.1152/ajpheart.00157.2013

[12] Ghio S , Guazzi M , Scardovi AB , Klersy C , Clemenza F , Carluccio E , Temporelli PL , Rossi A , Faggiano P , Traversi E , Vriz O , Dini FL , all investigators . Different correlates but similar prognostic implications for right ventricular dysfunction in heart failure patients with reduced or preserved ejection fraction. Eur J Heart Fail 2017; 19: 873–879. 27860029 10.1002/ejhf.664

[13] Felker GM , Anstrom KJ , Adams KF , Ezekowitz JA , Fiuzat M , Houston‐Miller N , Januzzi JL Jr , Mark DB , Piña IL , Passmore G , Whellan DJ , Yang H , Cooper LS , Leifer ES , Desvigne‐Nickens P , O'Connor CM . Effect of natriuretic peptide‐guided therapy on hospitalization or cardiovascular mortality in high‐risk patients with heart failure and reduced ejection fraction: a randomized clinical trial. JAMA 2017; 318: 713–720. 28829876 10.1001/jama.2017.10565 PMC5605776

[14] Ter Maaten JM , Valente MA , Damman K , Cleland JG , Givertz MM , Metra M , O'Connor CM , Teerlink JR , Ponikowski P , Bloomfield DM , Cotter G , Davison B , Subacius H , van Veldhuisen DJ , van der Meer P , Hillege HL , Gheorghiade M , Voors AA . Combining diuretic response and hemoconcentration to predict rehospitalization after admission for acute heart failure. Circ Heart Fail 2016; 9 e002845. 27266853 10.1161/CIRCHEARTFAILURE.115.002845

[15] Felker GM , Lee KL , Bull DA , Redfield MM , Stevenson LW , Goldsmith SR , LeWinter MM , Deswal A , Rouleau JL , Ofili EO , Anstrom KJ , Hernandez AF , McNulty SE , Velazquez EJ , Kfoury AG , Chen HH , Givertz MM , Semigran MJ , Bart BA , Mascette AM , Braunwald E , O'Connor CM , NHLBI Heart Failure Clinical Research Network . Diuretic strategies in patients with acute decompensated heart failure. N Engl J Med 2011; 364: 797–805. 21366472 10.1056/NEJMoa1005419 PMC3412356

